# adabmDCA: adaptive Boltzmann machine learning for biological sequences

**DOI:** 10.1186/s12859-021-04441-9

**Published:** 2021-10-29

**Authors:** Anna Paola Muntoni, Andrea Pagnani, Martin Weigt, Francesco Zamponi

**Affiliations:** 1 Statistical Inference and Biological Modeling Group, Italian Institute for Genomic Medicine, Candiolo, Italy; 2 Department of Applied Science and Technology, Politecnico di Torino, Turin, Italy; 3 Sezione di Torino, Istituto Nazionale Fisica Nucleare, Turin, Italy; 4 Institut de Biologie Paris Seine, Biologie Computationnelle et Quantitative LCQB, CNRS, Sorbonne Université, Paris, France; 5 Laboratoire de Physique de l’Ecole Normale Supérieure, ENS, CNRS, Université PSL, Sorbonne Université, Université de Paris, Paris, France

**Keywords:** Boltzmann machine learning, Protein modelling, RNA modelling, Statistical inference

## Abstract

**Background:**

Boltzmann machines are energy-based models that have been shown to provide an accurate statistical description of domains of evolutionary-related protein and RNA families. They are parametrized in terms of local biases accounting for residue conservation, and pairwise terms to model epistatic coevolution between residues. From the model parameters, it is possible to extract an accurate prediction of the three-dimensional contact map of the target domain. More recently, the accuracy of these models has been also assessed in terms of their ability in predicting mutational effects and generating *in silico* functional sequences.

**Results:**

Our adaptive implementation of Boltzmann machine learning, adabmDCA, can be generally applied to both protein and RNA families and accomplishes several learning set-ups, depending on the complexity of the input data and on the user requirements. The code is fully available at https://github.com/anna-pa-m/adabmDCA. As an example, we have performed the learning of three Boltzmann machines modeling the Kunitz and Beta-lactamase2 protein domains and TPP-riboswitch RNA domain.

**Conclusions:**

The models learned by adabmDCA are comparable to those obtained by state-of-the-art techniques for this task, in terms of the quality of the inferred contact map as well as of the synthetically generated sequences. In addition, the code implements both equilibrium and out-of-equilibrium learning, which allows for an accurate and lossless training when the equilibrium one is prohibitive in terms of computational time, and allows for pruning irrelevant parameters using an information-based criterion.

## Background

### Protein and RNA sequence modelling

In recent years, the number of available protein and RNA sequences has shown an impressive growth thanks to the development of high-throughput sequencing techniques. As a consequence, databases like Pfam [1] and Rfam [2], where such biological sequences are annotated and classified according to evolutionary relationships, play a dominant role in modeling this enormous amount of data.

In spite of this constant increase of sequence data, the tertiary structure of the corresponding domains is nowadays not experimentally accessible for the majority of the known protein and RNA sequences. To compensate for this experimental gap, *in silico* protein and RNA domains modeling has shown an incredible predictive power in determining their structure [3, 4]. An interesting way to achieve this goal is to perform data-driven modeling to reproduce some relevant statistical properties of the data (observables).

The so-called Direct Coupling Analysis (DCA) [5, 6] turns out to be particularly successful in using available homologous sequence data to infer structural determinants of the underlying protein or RNA domains [7]. In a nutshell, the DCA inference strategy provides a simple and informative interpretation of the inferred set of model parameters in terms of remarkably accurate contact map prediction. Among the different DCA implementations, Boltzmann machine learning [8–11] turns out to be one of the most efficient in terms of: (1) the accuracy of structural predictions in its direct use, or as input of more complex deep learning supervised strategies [12–15]; (2) the effectiveness to generate artificially-designed sequences that fold similarly to their natural counterparts [16]; (3) the ability to predict mutational effects [17, 18]. However, the quality of the Boltzmann machine deeply depends on the quality of the learning, which is intrinsically linked to the way the model observables are computed within the training, usually employing a Monte Carlo Markov Chain (MCMC).

Here, we present adabmDCA, an adaptive Boltzmann machine learning computational strategy that, taking as input a multiple sequence alignment of a target protein or RNA domain, infers efficiently an accurate statistical model of the sequence data with the twofold aim of (1) providing an accurate contact map prediction of the target domain, and (2) generating artificial sequences that are statistically close to indistinguishable (and thus *bona fide* biologically functional) from the natural. The code implements the so-called Boltzmann machine learning algorithm [19] by performing a gradient ascent of the *a posteriori* probability of the model given the input data. At variance with other implementations, adabmDCA copes with both protein and RNA data, and encompasses the ability of pruning redundant parameters (as described in [11]). Finally, it provides a standard *equilibrium* learning and also a *non-equilibrium* learning, based on the contrastive divergence technique [20], which is more suitable for structured data.

### An introduction to Boltzmann learning of biological models

The input of Boltzmann learning is a multiple sequence alignment (MSA) of a protein or a RNA family. The MSA contains *M* aligned sequences of length *L* of the same domain family, characterized by a certain structure and/or function. The key idea behind DCA is that evolutionarily related sequences show, together with a strong conservation signal on key residues (for instance in presence of active sites), large correlations among pairs of residues due to (mostly) structural constraints. If a random mutation affects the residue present on a certain site *i*, a compensatory mutation may be needed to appear in sites *j* in contact with residue *i* in order to preserve the overall structure of the domain. However, correlations alone provide poor results in terms of contact prediction (due to the presence of spurious correlations among sites not in direct contact), and therefore more sophisticated techniques such as DCA have been used to reliably extract the underlying coevolutionary signal.

In the following, we assume that each natural sequence $\varvec{s}=(s_1,\ldots ,s_L)$ belonging to a protein or RNA family is an independent and identically distributed (i.i.d.) sample of an unknown distribution (correlations due to phylogeny will be addressed through a standard re-weighting scheme explained below). Each residue $s_{i}$ takes value from an alphabet of *q* symbols representing all possible amino-acids or nucleic acids plus a ‘-’ gap symbol ($q = 21$ in the case of protein sequences or $q = 5$ for RNA). Given a MSA, we aim at finding a probability measure over sequences of length *L* that is able to accurately reproduce a set of chosen observables computed from the data. In particular, Boltzmann learning aims at reproducing all (or a subset of) the one-site and two-site empirical frequency counts. By applying the maximum entropy principle  [21], the least-constrained distribution that characterizes our data is a Boltzmann distribution:

1 $$\begin{aligned} P\left( \varvec{s}\right| \mathbf {J}, \mathbf {h}) = \frac{1}{Z\left( \mathbf {J}, \mathbf {h} \right) } \exp \left( \sum _{i} h_i\left( s_i\right) + \sum _{i<j} J_{i,j}\left( s_{i}, s_{j}\right) \right) \,, \end{aligned}$$

where *Z* is the normalization factor (the so called partition function in statistical-physics terminology) ensuring the proper normalization of the distribution, and $\mathbf {J}$ and $\mathbf {h}$ are the set of Lagrange multipliers assuring the fit of the moments, or, from a statistical physics point-of-view, the coupling matrices and the fields associated with the Potts model in Eq. 1. This probability quantifies the likelihood that a candidate sequence $\mathbf {s}$ belongs to the protein/RNA family characterized by the set of parameters $\mathbf {J},\mathbf {h}$. However, DCA aims at solving the related *inverse* problem: given a family’s MSA, how to determine the set of unknown parameters?

From a Bayesian perspective, the inverse problem can be mapped into the problem of finding the set of couplings $\mathbf {J}$ and fields $\mathbf {h}$ which maximize the posterior probability of the unknown parameters given the set of observed configurations of natural sequences

2 $$\begin{aligned} P\left( \mathbf {J},\mathbf {h}|\left\{ \varvec{s}^{\mu }:\mu =1,\ldots ,M\right\} \right) =\frac{P\left( \left\{ \varvec{s}^{\mu }\right\} |\mathbf {J},\mathbf {h}\right) P\left( \mathbf {J},\mathbf {h}\right) }{P\left( \left\{ \varvec{s}^{\mu }\right\} \right) }, \end{aligned}$$

where $P\left( \left\{ \varvec{s}^{\mu }\right\} \right)$ is the evidence (since it is independent of the unknown parameters, it will be neglected in the following), $P\left( \left\{ \varvec{s}^{\mu }\right\} |\mathbf {J},\mathbf {h}\right)$ is the likelihood function, which describes how probable it is to randomly draw the sequences in the MSA from the distribution parametrized by $\left( \mathbf {J},\mathbf {h}\right)$; and $P\left( \mathbf {J}, \mathbf {h} \right)$ is the prior probability distribution over the space of parameters. Recalling that sequences are i.i.d., the likelihood factorizes over sequences:

3 $$\begin{aligned} P\left( \left\{ \varvec{s}^{\mu }\right\} |\mathbf {J},\mathbf {h}\right)= & {} \prod _{\mu =1}^{M}P\left( \varvec{s}^{\mu }|\mathbf {J},\mathbf {h}\right) \end{aligned}$$

4 $$\begin{aligned}= & {} Z^{-M}\left( \mathbf {J},\mathbf {h}\right) \prod _{\mu }e^{\sum _{i=1}^{L}h_{i}\left( s_{i}^{\mu }\right) +\sum _{i<j}J_{ij}\left( s_{i}^{\mu },s_{j}^{\mu }\right) }\,. \end{aligned}$$

The prior $P\left( \mathbf {J},\mathbf {h}\right)$ provides additional information on the unknown parameters and it is exploited to avoid over-fitting. Indeed, very often a fully connected model (i.e. a model for which all couplings $\mathbf {J}$ are in principle different from 0) produces an over-parametrization of the unknown distribution as signaled by a large amount of noisy and negligible coupling parameters [11]. A practical way to control this behavior is to impose a sparsity prior over the coupling matrices: the two most used priors are the so called $\ell _{1}$ and $\ell _{2}$ regularizations, which force the inferred couplings to minimize the associated $\ell _{1}$ and $\ell _{2}$ norms multiplied by a tunable parameter $\lambda$ that sets the regularization strength. A complementary approach consists in setting *a priori* a probable topology suggested by the mutual information between all pairs of residues [22]. Here, as discussed in the following section, we will follow an information-based decimation protocol originally proposed in [11].

To set the stage, we first start by discussing the case, in which no prior information is considered. The maximization of the posterior distribution then turns out to be equivalent to the maximization of the likelihood function, or, equivalently, to the log-likelihood:

5 $$\begin{aligned} \mathcal {L}\left( \left\{ \varvec{s}^{\mu }\right\} |\mathbf {J},\mathbf {h}\right) =\frac{1}{M}\sum _{\mu }\left[ \sum _{i=1}^{L}h_{i}\left( s_{i}^{\mu }\right) +\sum _{i<j}J_{ij}\left( s_{i}^{\mu },s_{j}^{\mu }\right) \right] -\log Z\left( \mathbf {J},\mathbf {h}\right) \,. \end{aligned}$$

It is easy to prove that the log-likelihood is a globally convex function of the unknown parameters, hence a simple gradient ascent strategy is in principle able to find the optimal set of parameters. More precisely, starting from any initial guess for the parameters $\left\{ \mathbf {J}^{t=0},\mathbf {h}^{t=0}\right\}$, one can set up the following update scheme:

6 $$\begin{aligned} h_{i}^{t+1}\left( a\right)\leftarrow & {} h_{i}^{t}\left( a\right) + \eta _{h} \frac{\partial \mathcal {L}\left( \{\varvec{s}^{\mu }\} | \mathbf {J}^{t}, \mathbf {h}^{t}\right) }{\partial h_{i}\left( a\right) } \ , \end{aligned}$$

7 $$\begin{aligned} J_{ij}^{t+1}\left( a,b\right)\leftarrow & {} J_{ij}^{t}\left( a,b\right) + \eta _{J} \frac{\partial \mathcal {L}\left( \{\varvec{s}^{\mu }\} | \mathbf {J}^{t}, \mathbf {h}^{t}\right) }{\partial J_{ij}\left( a,b\right) } \ , \end{aligned}$$

until a fixed point is reached. Here, $\eta _{h}$ and $\eta _{J}$ are the learning rates associated with the fields $\mathbf {h}$ and the coupling parameters $\mathbf {J}$ respectively. A simple computation shows that the gradient terms involve averages of simple observables over the Boltzmann measure Eq. 1 with parameters at iteration time *t*:

8 $$\begin{aligned} \frac{\partial \mathcal {L}\left( \{\varvec{s}^{\mu }\} | \mathbf {J}^{t}, \mathbf {h}^{t}\right) }{\partial h_{i}\left( a\right) }= & {} f_{i}\left( a\right) - p_{i}^{\left( t\right) }\left( a\right) \ , \end{aligned}$$

9 $$\begin{aligned} \frac{\partial \mathcal {L}\left( \{\varvec{s}^{\mu }\} | \mathbf {J}^{t}, \mathbf {h}^{t}\right) }{\partial J_{ij}\left( a,b\right) }= & {} f_{ij}\left( a,b\right) - p^{\left( t\right) }_{ij}\left( a,b\right) . \end{aligned}$$

The stationary point is reached when the left hand sides of the equations are zero, i.e. when the single and double residue empirical frequency counts (i.e. the terms $f_i(a)$ and $f_{ij}(a,b)$ resp.) match the one- and two-site marginals $p_i(a)$ and $p_{ij}(b)$ of the model *P*. A formal definition of these quantities will be given in the next sections. Unfortunately, in spite of the relatively simple structure of the model, we are not able to exactly compute the marginal probability distributions. A practical way to overcome this limitation is to estimate them at each step *t* of the iteration by using a MCMC algorithm as explained in the following section.

## Implementation

### Input data and pre-processing

adabmDCA takes as input a MSA in FASTA format of the target protein or RNA family. To reduce the effect of phylogenetic correlations, we re-weight the statistical significance of each sequence, penalizing highly similar sequences in the MSA, as originally presented in [23]. In practice, with each of the *M* sequences of the MSA we associate a statistical weight $w^\mu$ ($\mu \in 1,\ldots ,M$) equal to the inverse number of sequences having at least 80% of identical residues with sequence $\mu$ (including sequence $\mu$ itself).

To deal with unobserved (pairs of) symbols in one (or two) column(s) of the MSA, we add a small pseudo-count $\alpha$ to the empirical frequency counts. This prevents the emergence of infinitely large parameters (in absolute value) associated with vanishing empirical frequencies. Finally, the one- and two-site frequencies are given by:

10 $$\begin{aligned} f_{i}\left( a\right)= & {} \left( 1-\alpha \right) f^{\mathrm{data}}_{i}\left( a\right) + \frac{\alpha }{q} \ , \end{aligned}$$

11 $$\begin{aligned} f_{ij}\left( a,b\right)= & {} \left( 1-\alpha \right) f^{\mathrm{data}}_{ij}\left( a,b\right) + \frac{\alpha }{q^{2}}\,, \end{aligned}$$

where $f^{\mathrm{data}}_{i}$ and $f^{\mathrm{data}}_{ij}$ are computed from the MSA as:

12 $$\begin{aligned} f^{\mathrm{data}}_{i}\left( a\right)= & {} \frac{1}{M_{\rm eff}}\sum _{\mu } w^{\mu } \delta _{s_{i}^{\mu },a} \ , \end{aligned}$$

13 $$\begin{aligned} f^{\mathrm{data}}_{ij}\left( a,b\right)= & {} \frac{1}{M_{\rm eff}} \sum _{\mu } w^{\mu } \delta _{s_{i}^{\mu },a} \delta _{s_{j}^{\mu },b}\,, \end{aligned}$$

with $M_{\rm eff} = \sum _\mu w^\mu$ being the effective number of weighted sequences.

### Initialization

In adabmDCA, it is possible to initialize the set of parameters in three ways: (1) all couplings and fields can be initially set to zero, (2) they can take value from a given set of parameters (from an input file), or (3) they describe a profile model, i.e. an independent-site Potts model where the first empirical moments are perfectly matched by means of the fields

14 $$\begin{aligned} h_{i}^{\mathrm{prof}}\left( a\right) = \log \left[ f_{i}\left( a\right) \right] + {\mathrm{const}}, \end{aligned}$$

but all couplings are set to zero. Empirically, it turns out that choice (3) is the one that shows the fastest convergence of the algorithm. We also allow for the other two types of initializations as they can be convenient in some cases.

### Adaptive Monte Carlo Markov Chain

The Boltzmann learning algorithm consists of a series of training epochs. At each epoch *t*, we estimate numerically the marginal probability distributions of the model $p^{(t)}_i(a)$ and $p^{(t)}_{ij}(a,b)$ using a MCMC strategy. More precisely, we use $N_{s}$ independent Markov chains, each of which samples $N_{c}$ configurations. The results presented in this work are obtained using a Metropolis-Hasting [24, 25] update scheme, but the code also allows one to opt for a Gibbs sampling strategy [26]. At the end of each epoch *t*, we update the model parameters according to Eq. 9 by estimating the $p^{(t)}_i(a), p^{(t)}_{ij}(a,b)$ according to the following relation:

15 $$\begin{aligned} p_{i}^{\left( t\right) }\left( a\right)&= \frac{1}{N_{s}N_{c} }\sum _{\mu =1}^{N_{s}N_{c}} \delta _{s_{i}^{\mu }\left( t\right) , a} \ , \end{aligned}$$

16 $$\begin{aligned} p_{ij}^{\left( t\right) }\left( a,b\right)&= \frac{1}{N_{s}N_{c}} \sum _{\mu =1}^{N_{s}N_{c}} \delta _{s_{i}^{\mu }\left( t\right) ,a}\delta _{s_{j}^{\mu }\left( t\right) ,b}\,. \end{aligned}$$

adabmDCA allows one to use either *persistent* chains, i.e. chains initialized only at the first epoch, or *transient* chains where each independent chain is initialized at each epoch. We consider two types of chain initialization: (1) by extracting sequences uniformly at random, (2) by randomly picking natural sequences from the MSA, proportionally to their weights $\varvec{w}$.

By default, adabmDCA uses *transient* chains initialized to uniformly extracted random sequences, but different options can be set. In particular, we found that the *persistent* option seems to reduce the equilibration time as one may expect that an equilibrium configuration extracted from the model at time $t-1$ is a good candidate starting point for the same chain at time *t* provided that the value of the parameters at time *t* is not too different from that at $t-1$.

In order to achieve accurate learning, it is of utmost importance to accurately estimate the gradient of the log-likelihood. From a computational point of view, the bottleneck is the accurate estimation of the one- and two-site marginals $p_i(a)$ and $p_{ij}(a,b)$. Two main conditions dictate the quality of MCMC sampling: (1) an accurate assessment of the stationary (i.e. equilibrium) regime of the chain, and, (2) a fair estimate of the mixing time.^1^ To prevent the occurrence of a poor sampling, adabmDCA allows for monitoring and adjusting both the equilibration and sampling times of the Markov Chain, *T*_eq_ and *T*_wait_, respectively (in Monte Carlo sweeps units, one sweep being equal to *L* Monte Carlo steps).

Let $\varvec{s}^{i}_{n}$ be the configuration sampled by chain *i* after $T_{\rm eq} + n T_{\rm wait}$ steps. We define three type of sequence identities or *overlaps*, i.e.

17 $$\begin{aligned} O\left( \varvec{s}^{i}_{n}, \varvec{s}^{k}_{n}\right) := \sum _{j=1}^{L}\delta _{s^{i}_{n}\left( j\right) , s^{k}_{n}\left( j\right) } , \end{aligned}$$

aimed at quantifying how similar two target configurations are:

- The *external* overlap between configurations sampled by two different chains at the same sampling time *n*
  $$\begin{aligned} Q^{\rm ext}\left( i,k,n\right) = O\left( \varvec{s}^{i}_{n}, \varvec{s}^{k}_{n}\right) \, {\mathrm {for}}\, i\ne k. \end{aligned}$$
- A *first-time internal* overlap measuring the similarity between two consecutively sampled configurations on the same chain:
  $$\begin{aligned} Q^{\rm int1}\left( i,n,n+1\right) = O\left( \varvec{s}^{i}_{n}, \varvec{s}^{i}_{n+1}\right). \end{aligned}$$
- A *second-time internal* overlap measuring the distance between configuration sampled at time *n* and $n+2$ on the same chain:
  $$\begin{aligned} Q^{\rm int2}\left( i,n,n+2\right) = O\left( \varvec{s}^{i}_{n},\varvec{s}^{i}_{n+2}\right). \end{aligned}$$

At each iteration, we compute the expectation value $\mu _{\alpha }$ and the standard error $\sigma _{\alpha }$ (where the averages are computed with respect to different chains and over *n*) of $Q^{\alpha }$ for all three types of overlap $\alpha \in \{{\mathrm{int1~int2~ext}}\}$. We note that, if *T*_eq_ and *T*_wait_ were large enough, then subsequent samples of the same chain should have the same statistics of samples coming from distinct chains, and $\mu _{\rm ext} = \mu _{\rm int1} = \mu _{\rm int2}$ within statistical errors. Therefore, we update *T*_wait_ as follows:

- If $|\mu _{\rm ext} - \mu _{\rm int2}| > 5 \sqrt{\sigma ^{2}_{\rm ext} + \sigma ^{2}_{\rm int2}}$ we say that our Monte Carlo chains are not sufficiently de-correlated and therefore we increase *T*_wait_.
- Conversely, if $|\mu _{\rm ext} - \mu _{\rm int1}| < 5 \sqrt{\sigma ^{2}_{\rm ext} + \sigma ^{2}_{\rm int1}}$ the chains sufficiently de-correlate every *T*_wait_ steps and, as a consequence, we can reduce *T*_wait_.

This allows the chains to be slightly correlated at time *T*_wait_ but ensures a good de-correlation at time 2*T*_wait_, hence guaranteeing that the de-correlation time is in between *T*_wait_ and 2*T*_wait_. To increase *T*_wait_, we double it, while to reduce *T*_wait_, adabmDCA computes the average between the current value of the waiting time and the value of *T*_wait_ before the last increasing step. This guarantees to keep the waiting time bounded in the correct interval of values within the learning process. Then, whatever the outcome of this test, we set *T*_eq_ = 2*T*_wait_ assuming that 2*T*_wait_ steps suffice to get equilibrated samples starting from the first configuration of the chain. Note that when the starting sample is picked uniformly at random, this criterion does not guarantee a perfect equilibration because the equilibration time might be in some cases larger than the de-correlation time, although this is expected to happen rarely; conversely, for persistent chains, this condition guarantees equilibration by construction, because in that case the chains do not need to be re-equilibrated at each iteration.

When *T*_wait_ and *T*_eq_ are such that $\mu _{ext} \sim \mu _{int2}$, adabmDCA achieves a well-equilibrated sampling and the Boltzmann machine is guaranteed to converge to a Potts model, which not only precisely fits the one- and two-site frequencies, but benefits of several additional properties elaborated in the Results section. However, depending on the properties of the data, several issues can arise: if the true energy landscape is sufficiently rugged, Monte Carlo chains may partially visit the feasible configurations returning a sampling that strongly depend on the initialization of the chains. Similarly, if the model parameters are abruptly adjusted, the dynamic may mimic a low-temperature regime of a well-behaved landscape ending up to the same sampling issue of the rugged energy landscape. In both cases, the dynamics becomes non-ergodic, and therefore the computation of the gradient may be inaccurate. For this reason a smooth update of the parameters is encouraged and, in cases when this is not sufficient, we found that, using persistent chains with fixed (but large) sampling time, adabmDCA performs equally well. In this scenario, even though the machine performs the sampling using slightly correlated chains, the quality of the inferred model is often not affected. We show an example in the Results section.

### Convergence criterion and quality control

Given the global convexity of the problem as a function of the parameters, the convergence of the algorithm can be safely assessed when the gradients are numerically close to zero. A convenient proxy for convergence is given by the difference between the empirical and the model two-site connected correlations (or co-variances):

18 $$\begin{aligned} c_{ij}^{\rm model} \left( a,b\right)&= p_{ij} \left( a,b\right) - p_{i}\left( a\right) p_{j}\left( b\right), \end{aligned}$$

19 $$\begin{aligned} c_{ij}^{\rm emp} \left( a,b\right)&= f_{ij} \left( a,b\right) - f_{i}\left( a\right) f_{j}\left( b\right). \end{aligned}$$

The learning halts when the tolerance $\varepsilon _{c} = \arg \max _{i,j,a,b} | \, c_{ij}^{\rm model}\left( a,b\right) - c_{ij}^{\rm emp}\left( a,b\right) \, |$ is $\sim 10^{-2}$. Although this quantity is not explicitly fitted during learning, it is a function of the one- and two-site frequencies in Eqs. 8, 9 and vanishes at convergence. Empirically, it provides a good metric for estimating the quality of the inferred model. At each iteration, we also measure the Pearson correlation coefficient between the empirical and model covariances defined in Eqs. 18, 19, which measures a degree of correlation between the two quantities independently of the value of $\varepsilon _{c}$, i.e. of the spread of the scatter plot of the connected covariance. Moreover, we display the fitting error of the one- and two-site statistics computed as $\varepsilon _{f} = \sum _{i,a} \frac{| f_{i}\left( a\right) - p_{i} \left( a\right) |}{Lq}$, $\varepsilon _{s} = \sum _{i,j,a,b} \frac{| f_{ij} \left( a, b \right) - p_{ij} \left( a,b \right) |}{L^2q^2}$ ; these metrics indeed help in monitoring the training of the Boltzmann machine.

Another interesting observable that can be used to assess the generative power of the Boltzmann machine, is the three-site connected correlation

$$\begin{aligned} {\begin{matrix} c_{ijk}(a,b,c)&:=f_{ijk}(a,b,c)-f_{ij}(a,b)f_k(c)-f_{ik}(a,c)f_j(b)\\ &-f_{jk}(b,c)f_i(a)+2f_i(a)f_j(b)f_k(c) \ , \end{matrix}} \end{aligned}$$

which is not fitted during the training but, as shown in [10, 11], provides an interesting measure of the generative capability of the model. adabmDCA does not compute all possible third order connected correlations because this would be computationally heavy. However, it is possible to specify a subset of indices $\left( i,j,k\right)$ and $\left( a,b,c\right)$ whose corresponding measures are computed during the iterations.

### Gauge fixing

The number of unknown parameters $N_{p} = \frac{L\left( L-1\right) }{2}q^{2}+Lq$ exceeds the number of independent Eqs. 8, 9 (when setting the partial derivatives to zero), due to the normalization constraint on the one-site and two-site statistics, $\sum _{a}f_{i}\left( a\right) = 1$, $\sum _{a,b} f_{ij}\left( a,b\right) = 1$ and the marginalization condition over the two-site statistics, $\sum _{a}f_{ij}\left( a,b\right) = f_{i}\left( b\right)$. As a consequence, any *gauge* transformation of the type

20 $$\begin{aligned} J_{ij}\left( a,b\right)\rightarrow & J_{ij}\left( a,b\right) + K_{ij}\left( a\right) + K_{ji}\left( b\right) \end{aligned}$$

21 $$\begin{aligned} h_{i}\left( a\right)\rightarrow & h_{i}\left( a\right) + g_{i} - \sum _{j\ne i}\left[ K_{ij}\left( a\right) + K_{ji}\left( a\right) \right] \end{aligned}$$

for arbitrary $g_{i}$ and $K_{i,j}\left( a\right)$, would keep unchanged the Boltzmann distribution in Eq. 1. Among the infinite number of possible *gauge* transformations, the one of most interest is the so-called *zero-sum* gauge because the couplings obtained by this re-parametrization minimize the Frobenius norms associated with the coupling matrices. This transformation is applied at the end of the Boltzmann machine learning to facilitate the computation of the DCA scores.

Alternatively, one may fix the *gauge* at the beginning of the learning, by fixing a redundant subset of the parameters to an arbitrary constant and then update the remaining parameters within the learning. To select the redundant subset, for each couple $\left( i,j\right)$, we seek the $2q-1$ pairs of colors that give the weakest empirical connected correlations, computed as in Eq. 19, and we set to zero the couplings associated with these variables. These couplings are fixed to zero also during learning.

### Pruning the parameters

Although the gauge fixing removes the degree of variability of the inferred parameters, due to the finite sample size of the MSA, the trained model might still be over-fitted. Indeed, sequence lengths *L* in typical MSA range in the interval $\sim 100 - 500$. As a consequence, the number of learned parameters is $\sim 10^{7}-10^{9}$, which likely exceeds the useful information encoded in the data. A widely used strategy to limit over-fitting is to impose an $\ell _{1}$ or $\ell _{2}$ regularization, i.e. a prior, either to both the set of parameters or to the couplings only. In these cases, the update Eqs. 8–9 are replaced by the gradient of the log-posterior:

22 $$\begin{aligned} \frac{\partial \mathrm {log}P \left( \mathbf {J}^{t}, \mathbf {h}^{t} | \{\varvec{s}^{\mu }\} \right) }{\partial h_{i}\left( a\right) }= & {} f_{i}\left( a\right) - p_{i}\left( a\right) -\lambda _{1} \mathrm{sign} \left[ h_{i}\left( a\right) \right] \ , \end{aligned}$$

23 $$\begin{aligned} \frac{\partial \mathrm {log} P \left( \mathbf {J}^{t}, \mathbf {h}^{t} | \{\varvec{s}^{\mu }\} \right) }{\partial J_{ij}\left( a,b\right) }= & {} f_{i,j}\left( a,b\right) - p_{i,j}\left( a,b\right) - \lambda _{1} \mathrm{sign} \left[ J_{i,j}\left( a,b\right) \right] , \end{aligned}$$

and

24 $$\begin{aligned} \frac{\partial \mathrm {log} P \left( \mathbf {J}^{t}, \mathbf {h}^{t} | \{\varvec{s}^{\mu }\} \right) }{\partial h_{i}\left( a\right) }&= f_{i}\left( a\right) - p_{i}\left( a\right) -\lambda _{2} h_{i}\left( a\right) \ , \end{aligned}$$

25 $$\begin{aligned} \frac{\partial \mathrm {log} P \left( \mathbf {J}^{t}, \mathbf {h}^{t} | \{\varvec{s}^{\mu }\} \right) }{\partial J_{ij}\left( a,b\right) }&= f_{i,j}\left( a,b\right) - p_{i,j}\left( a,b\right) - \lambda _{2} J_{i,j}\left( a,b\right) \ , \end{aligned}$$

for the $\ell _{1}$ and $\ell _{2}$ priors respectively.

The main drawback of these procedures is that the regularization is applied indistinctly to all parameters (relevant and irrelevant). Alternatively, one may *a priori* specifically prune (viz. set to zero) a subset of the parameters observing that even though large spurious correlations may arise from non topologically connected sites, weak correlations are typically associated with small coupling strengths. As explained in [22], one can first determine a starting topology and then run the learning procedure on it. To this end, adabmDCA provides two distinct strategies. Indeed, the user can:

- provide as input a given topology (i.e. a set of predefined pairs of residues that will not be set to 0); adabmDCA then automatically eliminates all absent parameters before the learning;
- iteratively remove negligible couplings up to a target sparsity as explained in [11]. To determine whether a coupling matrix (or element) is negligible, we compute the symmetric Kullback-Leibler divergence between the model at the current time-step and the same model without that coupling matrix (or element). The latter is used to score the parameters and set to zero those with the smallest score. The parameter is set to zero *element-wise* if we remove negligible couplings drawn on different matrices or *block-wise* if we remove an entire $\mathbf {J}_{ij}$ matrix. We refer to [11] for details of the element-wise decimation.

### Adaptive learning rate

The learning rates $\eta _{J}$ and $\eta _{h}$ associated with the update of the fields and couplings, respectively, are set by default to a small and constant value, typically 0.05 for proteins and 0.01 for RNA families. Alternatively, several adaptive learning rates can be used to train the Boltzmann machine: *adagrad* [27], *search-then-converge* [28], a modified *quasi-Newton* method [29–31] and *FIRE* [32]. Although using an adaptive learning rate allows for a fast training of the parameters (as indicated by a rapid increasing of the Pearson correlation coefficient between the data and model covariances already in the first few iterations), the possibly large learning rates push the value of the parameters to large (absolute) values preventing a good equilibration of the machine within the training, and often resulting in over-fitting.

### Schematic workflow

**Fig. 1:**
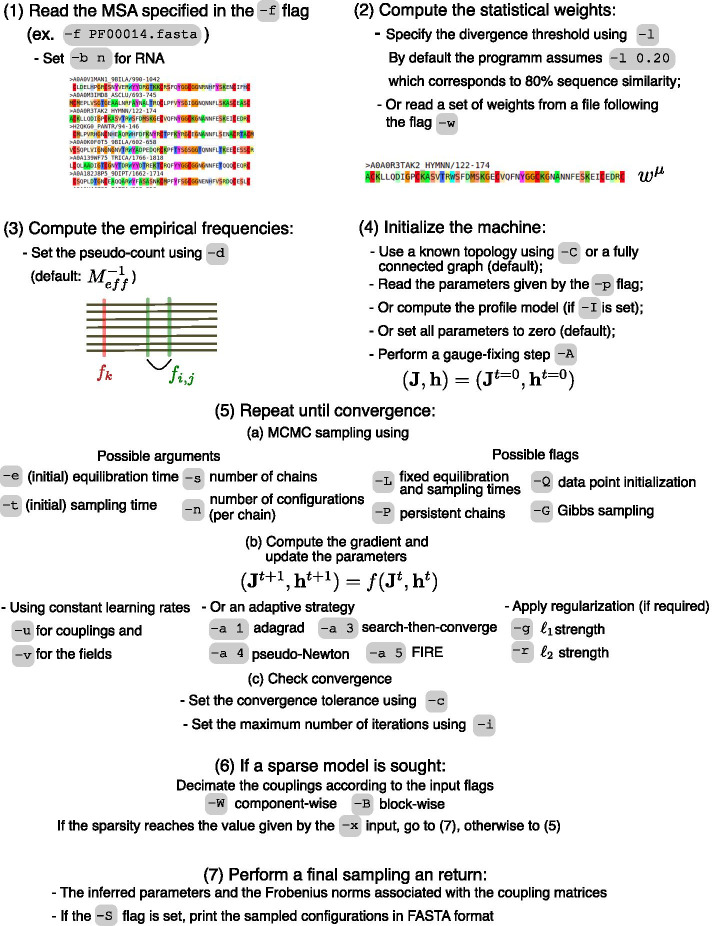
Workflow of adabmDCA. We show a schematic road-map of the main adabmDCA workflow specifying the input flags associated with the possible tasks that can be executed by the algorithm. Lower case flags are usually followed by an argument whereas upper case flags appear alone

To clarify the main adabmDCA road-map we plot in Fig.  1 a schematic representation of the features performed by the algorithm (as well as the most important input flags):

- *Reading the natural sequences.* The algorithm first reads a FASTA file containing the multiple sequence alignment of protein or RNA sequences.
- *Re-weighting of the sequences.* adabmDCA either takes as input a file storing the statistical weights of the sequences or it applies the re-weighting scheme explained in Section 2.1.
- *Computation of the observables.* Once the weights are computed, it is possible to evaluate and store the one-site and two-site frequencies appearing in the log-likelihood (or log-posterior) as in Eqs. 12–13. The pseudo-count $\alpha$ can be arbitrarily set or, by default, it takes the value of $M_{eff}^{-1}$.
- *Initialization of the machine.* By default, the machine assumes a fully connected model and the parameters are set to zero. Alternatively, a profile model can be chosen using a pre-defined flag or the machine can read an input set of parameters from a file. The gauge-fixing procedure, as explained in Sect. 2.5 can be performed using a specific flag. Furthermore, in cases where the topology is known, adabmDCA can read from files the (possibly non-zero) couplings and the fields of the machine and set permanently to zero the remaining part.
- *Update of the parameters until convergence.* At each epoch, adabmDCA performs a MCMC sampling as described in Sect. 2.3 to estimate the model statistics. All possible flags used to set up the MCMC sampling are shown in Fig. 1. By default, the equilibration and sampling times are adaptively tuned as described in Sect. 2.3. Then, the parameters are updated accordingly to the gradient as in Eqs. 6–7 (or as in Eqs. 22–23 or 24–25) depending on the presence (or absence) of the regularization terms. The learning rate is by default constant during the training but, if required by the user, several adaptive learning strategies are implemented (see Sect. 2.7).
- *Decimation.* If required and if convergence is reached, adabmDCA performs a component-wise or block-wise pruning of the coupling matrices according to an information-based criterium (see Sect. 2.6). Then, the algorithm alternates the convergence step to the pruning step of the Boltzmann machine until a converged model having the required density is sought.
- *Output of the results.* The algorithm performs a final sampling of the converged Boltzmann machine and prints in several files the couplings and fields of the model as well as the Frobenius norms, i.e. the Direct Coupling scores, associated with the $\mathbf {J}_{ij}$ matrices. If required by the user adabmDCA outputs the sampled configurations in FASTA format.

In the following, we report few examples to launch adabmDCA in some interesting cases, useful to reproduce the results of the following section:

- *Learning at equilibrium.* Let us train a Boltzmann machine for the sequences contained in file.fasta at equilibrium, starting from a profile model and requiring a tolerance of $10^{-2}$ for the two-site connected correlations, using our machine. The command line will read:
  26 $$\begin{aligned} \texttt {./adabmDCA -f file.fasta -I -c 1e-2} \end{aligned}$$
- *Learning out-of-equilibrium.* To use persistent chains and avoid the tuning of the MCMC characteristic times, we will add:
  27 $$\begin{aligned} \texttt {./adabmDCA -f file.fasta -I -c 1e-2 -L -P} \end{aligned}$$
- *Sampling.* Let us sample a given model stored in the file p.dat using $T_{eq} = 500$ and $T_{wait} = 250$. The command line reads:
  28 $$\begin{aligned} &\texttt {./adabmDCA -f file.fasta -p p.dat }\\ &\quad \texttt {-i 0-e 500 -t 250 -L -S} \end{aligned}$$

## Results

We now discuss some examples of model learning via adabmDCA on protein and RNA families.

### Learning at equilibrium: PF00014 and RF00059

In this section we show the results obtained for: (1) the Kunitz domain (PF00014 family from the Pfam database), (2) the TPP riboswitch (RF00059 from the Rfam database). The PF00014 MSA is initially pre-processed to remove from the MSA all proteins with more than six consecutive gaps. This prevents a learning bias towards very gapped configurations. Eventually, the total number of considered sequences is *M* = 13600 for PF00014 and *M* = 12593 for RF00059, which correspond to a re-weighted effective number of sequences of $M_{\rm eff} = 4364$ and $M_{\rm eff} = 4920$ for PF00014 and RF00059 respectively.

**Fig. 2:**
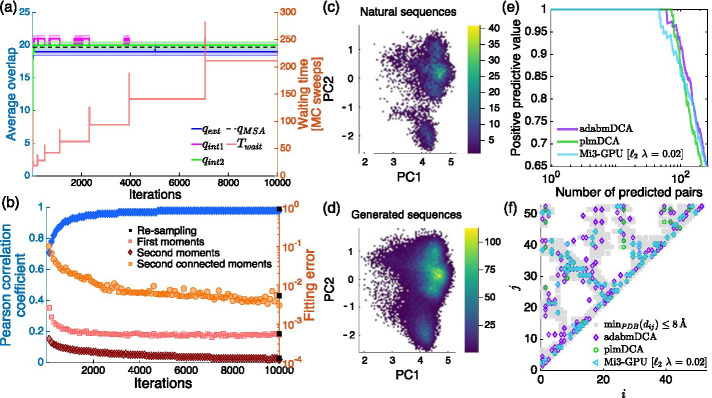
Learning of PF00014 at equilibrium We show in **a** the evolution over the iterations of the three overlaps used to monitor the quality of the sampling together with the waiting time *T*_wait_. **b** We plot, for all iterations, the fitting errors (red, orange and brown markers) associated with the one-site, two-site (connected and non-connected) frequencies computed as defined by $\varepsilon _{f}$, $\varepsilon _{s}$ and $\varepsilon _{c}$ in Sect. 2.4. Using a blue marker we show the Pearson correlation coefficient between the two-site connected frequencies of the natural sequences and of the configurations generated during training. **c** We plot the projections of the natural sequences into the space of the first two principal components (PC1, PC2) of the covariance matrix associated with the natural sequences while in **d** we project the configurations obtained by the re-sampling of the converged model into PC1 and PC2 associated with the natural sequences. **e** Depicts the behavior of the positive predictive value (PPV) versus the number of non-trivial contact predictions, i.e. those associated with site indices $|i-j|>4$, for adabmDCA, plmDCA [33] and Mi3-GPU [31]. **f** We instead plot the contact maps used for the comparison in **e**: gray blocks are associated with the ground-truth obtained by Pfam-interactions [35], while the colored markers indicate whether the Frobenius norms computed using the parameters retrieved by the three methods are larger than 0.20

**Fig. 3:**
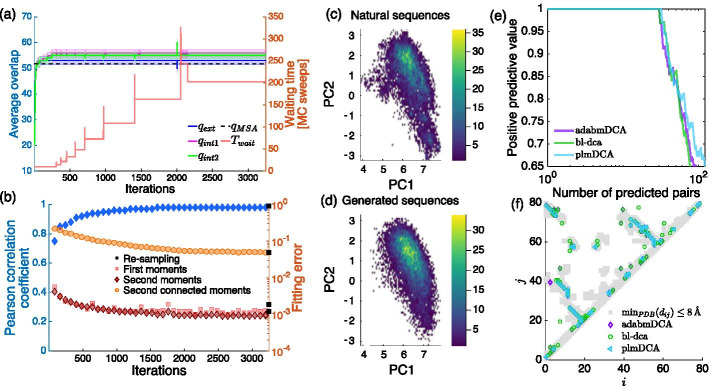
Learning of RF00059 at equilibrium. **a** The evolution over the iterations of $q_{ext}$, $q_{int1}$ and $q_{int2}$ used to tune the waiting time $T_{wait}$. **b** We plot the fitting errors (red, orange and brown markers) $\varepsilon _{c}$, $\varepsilon _{f}$ and $\varepsilon _{s}$, and the Pearson correlation coefficient between the two-site connected statistics of the natural sequences and of the configurations sampled during training (blue markers). **c** Depicts the projections of the natural sequences into the space of the first two principal components (PC1, PC2) of the covariance matrix associated with the natural sequences; in **d** we show the projection the re-sampled configurations, obtained from the converged model, into PC1 and PC2 associated with the natural sequences. **e** We show the behavior of the PPV versus the number of non-trivial contact predictions, i.e. those associated with site indices $|i-j|>4$, for adabmDCA, plmDCA [33] and bl-dca [34]. **f** Displays the contact maps used as ground truth (gray markers) for the TPP riboswitch and those obtained by the DCA scores larger than 0.20 associated with the three compared methods

The Boltzmann machines are trained at equilibrium, i.e. the waiting and equilibrium times are updated at each iteration according to the test introduced in Sect. 2.3. The behavior of the average of the three overlaps $q_{\alpha }$ for $\alpha = \{\mathrm{int1,~int2,~ext}\}$ is shown in Figs. 2a and 3a (left axis) together with the trend of the waiting time *T*_wait_ (right axis). One can see that the distribution of the mean for the three quantities show statistically compatible values. Interestingly, starting from the beginning of the training, their average value is very close to the mean overlap among all pairs of natural sequences used within the learning, shown as $q_{MSA}$ in the plot. The waiting time is typically increased at the beginning of the training and it seems to stabilize at the final iterations.

The quality of the Boltzmann machine is monitored during the learning as shown in Figs. 2b and 3b where we display the Pearson correlation coefficient between the model and the empirical connected two-site frequencies as computed in Eqs. 19 (left axis) as a blue line together with the mean error achieved in fitting the one-site and (connected or non-connected) two-site frequencies (right axis). At the final iteration we get a very accurate model as signaled by the high value of the Pearson correlation coefficient and the small values of the fitting errors, which are perfectly retrieved if one samples the final models using a very long Monte Carlo Markov Chain (black squared point), i.e. by imposing *T*_eq_ = 5000 and *T*_wait_ = 2500. The generative power of the Boltzmann machines is corroborated by comparing the Principal Component Analysis (PCA) of the generated sequences with the natural sequences as shown in Figs. 2, 3c, d respectively. The sampled configurations in panel (d) are projected onto the first two principal components of the natural sequences in panel (c). As suggested by the spatial localization of the sequences and their distribution, our converged models are able to generate sequences that lie in the same non-trivial sub-space spanned by the natural sequences.

Finally, we compare the predicted contact maps of the Kunitz domain and of the TPP riboswitch with the following state-of-the-art DCA-based algorithms: plmDCA [33] and Mi3-GPU [31] for PF00014 and bl-dca [34] for RF00059, one pseudo-likelihood method and two Boltzmann machine-based methods to infer Potts models for protein and RNA sequences respectively.

In all cases, coupling parameters are first converted to zero-sum gauge before computing the average product corrected Frobenius norms [33]. For PF00014, we consider as ground-truth the atomic distances retrieved by Pfam-interactions [35], a method which computes the minimum distance, for all possible pair of sites, among all available crystal structures in the Protein Data Bank (PDB). For RF00059 we perform an analogous analysis among the TPP riboswitch known structures downloaded from the Protein Data Bank. In Figs. 2, 3e we plot the positive predictive value of the prediction of the non-trivial contacts, i.e. those residue pairs (*i*, *j*) having $|i-j| > 4$, for the three methods, and in Figs. 2, 3f we overlap our ground-truth (in gray) and the most probable contact according to the three-methods, i.e. the pairs with whom we associate a score larger than 0.20. For Mi3-GPU we consider the model obtained applying an $\ell _{2}$ regularization with strength parameter $\lambda = 0.02$; the machine obtained for the $\ell _{1}$ regularization gives a dramatically worse results in terms of contact predictions (not shown). Panel (e) suggests that the three considered methods achieve comparable performances, as it is equivalently represented in panel (f).

### Learning out-of-equilibrium: PF13354

**Fig. 4:**
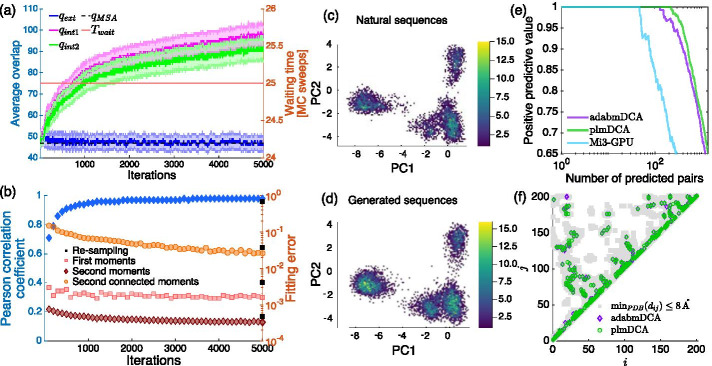
Learning of PF13354 out-of-equilibrium. **a** We show the three overlaps $q_{ext}$, $q_{int1}$ and $q_{int2}$ of the sampled configurations used to estimate the model statistics as a function of the iterations (left axis) and the waiting time *T*_wait_ between two consecutive samples (right axis). At difference with the learning at equilibrium, *T*_wait_ is here kept constant during the training and the configurations are correlated as suggested by the differences between the distributions of $q_{int1}$, $q_{int2}$ and $q_{ext}$. **b** The plot of the quality metrics used to estimate the goodness of the training: in blue we show the Pearson correlation coefficient between the two-site connected frequencies of the natural sequences and of the evolving model as a function of the iterations (blue markers, left axis) and the fitting errors (red, orange and brown markers, right axis) computed as $\varepsilon _{c}$ for the two-site connected statistics and as $\varepsilon _{f,s}$ for the one-site and two-site non-connected statistics. **c**, **d** We show the projections of the natural sequences and of the re-sampled sequences into the first two principal components of the natural sequences while in **e** we plot the positive predictive value curve associated with the contact map prediction (shown in **f**) for the Beta-lactamase2 domain

In this section we show the results obtained for the Beta-lactamase2 domain. The multiple sequence alignment used within the training is constructed as follows. Using the Hidden Markov Model associated with the PF13354 family, we scanned the NCBI [36] database to obtain aligned sequences compatible with the model. We then keep sequences that have less than 20% of gaps and concurrently those having less than 80% redundancy (as a consequence $M_{\rm eff} \sim M$ in this case). We also removed the sequence of the TEM-1 protein, and all sequences very similar to it. This last step was necessary to study deep mutational scanning data in [11] and we use here the same alignment for sake of simplicity. Training a Boltzmann machine using well-equilibrated Monte Carlo chains is barely practical as the waiting time necessary to produce uncorrelated samples is huge and constantly increasing over the iterations (not shown). To solve this issue, we resort to a *persistent* sampling strategy, i.e. at each new iteration the Monte Carlo chains are initialized at the last configurations of the previous iteration, of $10^{3}$ chains, each one sampling 10 configurations, with fixed waiting time $T_{\rm wait} = 25$ and equilibrium time $T_{\rm eq} = 50$ sweeps. In Fig. 4a we display the overlap between independent chains $q_{ext}$, which is similar to that of the MSA of the natural sequences $q_{MSA}$, while $q_{int1}$ and $q_{int2}$ grow over the iterations suggesting that the samples are highly correlated. The fitting quality of the model is measured by using the Pearson correlation coefficient (blue markers) and the fitting errors over the one-site and two-site statistics (red, orange and brown markers) as shown in Fig. 4b; these measures are compatible to those obtained by a learning at equilibrium. To check the quality of the learning out-of-equilibrium, we re-sample the converged model and test the generative properties of the learned machine. The Pearson correlation coefficient and the fitting errors of the converged model are retrieved only if the configurations obtained by the re-sampling step are sufficiently de-correlated: indeed, to obtain the performances shown using black markers in Fig. 4b one has to set $T_{\rm wait} \sim 10^{5}$ which is the value of the waiting time that guarantees $q_{int1} \sim q_{int2}$. The remarkable results of the Beta-lactamase2 model are confirmed by the PCA analysis in Fig. 4c, d and by the contact prediction depicted in panels (e) and (f). In this case, adabmDCA achieves a reconstruction similar to that of plmDCA and outperforms Mi3-GPU where the adaptive strategy to sample statistically independent equilibrium configurations fails to produce a result due to the too large auto-correlation time estimated.

This result suggests that although we are not able to achieve an equilibrium sampling due to the large auto-correlation time, yet the resulting model retains the generative properties of an equilibrium-trained Boltzmann machine. Not only this result is important from a practical point of view, as this allows for a significant reduction of the computational time of the overall process, but it also opens new research directions in the field of out-of-equilibrium learning. We mention that if the procedure is performed using randomly initialized chains, instead of persistent chains, the quality of the converged model is achieved only setting a waiting time similar to that used in the training, as if the model had kept memory of the learning set-up. A similar behavior has been observed and discussed more systematically in [37] in the context of learning Restricted Boltzmann machines.

### Running time

We discuss in this section the computation time of adabmDCA. The running time needed by adabmDCA is often larger than the ones shown by the other methods used here for comparison: our machine spent 22, 53, and 98 hours for learning a model for PF00014, RF00059, and PF13354, respectively, against one hour and the 75 hours required by Mi3-GPU for PF00014 and PF13354 (employing two TITAN RTX GPUs) and the two hours needed by bl-dca for RF00059. We stress that the current implementation exploits a single thread and its running times are compatible with those achieved by the Boltzmann machine in [10]. Moreover, the out-of-equilibrium learning allows for the training of an accurate machine, out-performing Mi3-GPU and spending a running time which is only slightly larger than that needed by Mi3-GPU, a highly optimized algorithm.

Fortunately, a multi-threads implementation of adabmDCA can be certainly attained by running in parallel the MCMC sampling, i.e. each thread could perform the simulations of a certain fraction of the MC chains, independently of the other threads. This direction will be considered in the future development of the algorithm.

## Conclusions

We developed a C/C++ implementation of Boltzmann machine learning for modeling RNA and protein sequence families, called adabmDCA. Together with a set of learning options that allows for a user-friendly control of the training strategy (including parameters initialization, regularization and decimation), it encompasses the possibility of adapting the Monte Carlo Markov Chain sampling ensuring an equilibrium training. In hard learning regimes, when the de-correlation time of the Monte Carlo chains appears to be large, the learning at equilibrium is intractable. In these cases, in adabmDCA it is possible to select a slightly out-of-equilibrium sampling whose behavior does not affect the quality of the learned model, as suggested by the results on the Beta-lactamase2 domain. Here, the performances of adabmDCA resemble those of plmDCA in predicting non-trivial physical contacts and outperforms other Boltzmann machine-like implementations. This promising achievement encourages new research perspectives in the field of non-equilibrium learning.

## Availability and Requirements

Project name: adabmDCA

Project home page: https://github.com/anna-pa-m/adabmDCA

Operating systems: Linux, Mac OS and Windows

Programming languages: C/C++

Licence: MIT Licence

Any restriction to use by non-academics: No.

## Data Availability

The multiple sequence alignments analysed during the current study are available in the GitHub repository https://github.com/anna-pa-m/adabmDCA.

## Abbreviations

DCA: Direct coupling analysis
PCA: Principal components analysis
MSA: Multiple sequence alignment
PPV: Positive predictive value
MCMC: Monte Carlo Markov Chain

## Footnotes

1 adabmDCA estimates the mixing time of the MCMC through the auto-correlation time of the sampled configurations

## References

[1] Mistry J, Chuguransky S, Williams L, Qureshi M, Salazar GA, Sonnhammer ELL, Tosatto SCE, Paladin L, Raj S, Richardson LJ, Finn RD, Bateman A (2021). Pfam: The protein families database in 2021. Nucleic Acids Res..

[2] Kalvari I, Nawrocki EP, Ontiveros-Palacios N, Argasinska J, Lamkiewicz K, Marz M, Griffiths-Jones S, Toffano-Nioche C, Gautheret D, Weinberg Z, Rivas E, Eddy SR, Finn R, Bateman A, Petrov AI (2021). Rfam 14: expanded coverage of metagenomic viral and microRNA families. Chem Rev.

[3] Jumper J, Evans R, Pritzel A, Green T, Figurnov M, Ronneberger O, Tunyasuvunakool K, Bates R, Žídek A, Potapenko A, et al. Highly accurate protein structure prediction with alphafold. Nature. 2021;1–11. 10.1038/s41586-021-03819-2. 10.1038/s41586-021-03819-2 PMC8371605 34265844

[4] Baek M, DiMaio F, Anishchenko I, Dauparas J, Ovchinnikov S, Lee GR, Wang J, Cong Q, Kinch LN, Schaeffer RD, Millán C, Park H, Adams C, Glassman CR, DeGiovanni A, Pereira JH, Rodrigues AV, van Dijk AA, Ebrecht AC, Opperman DJ, Sagmeister T, Buhlheller C, Pavkov-Keller T, Rathinaswamy MK, Dalwadi U, Yip CK, Burke JE, Garcia KC, Grishin NV, Adams PD, Read RJ, Baker D (2021) Accurate prediction of protein structures and interactions using a three-track neural network. Science eabj8754. 10.1126/science.abj8754 10.1126/science.abj8754 PMC7612213 34282049

[5] Weigt M, White RA, Szurmant H, Hoch JA, Hwa T (2009). Identification of direct residue contacts in protein–protein interaction by message passing. Proc Natl Acad Sci.

[6] Morcos F, Pagnani A, Lunt B, Bertolino A, Marks DS, Sander C, Zecchina R, Onuchic JN, Hwa T, Weigt M (2011). Direct-coupling analysis of residue coevolution captures native contacts across many protein families. Proc Natl Acad Sci.

[7] Cocco S, Feinauer C, Figliuzzi M, Monasson R, Weigt M (2018). Inverse statistical physics of protein sequences: a key issues review. Rep Prog Phys..

[8] Sutto L, Marsili S, Valencia A, Gervasio FL (2015). From residue coevolution to protein conformational ensembles and functional dynamics. Proc Nat Acad Sci..

[9] Haldane A, Flynn WF, He P, Vijayan RSK, Levy RM (2016). Structural propensities of kinase family proteins from a potts model of residue co-variation. Protein Sci..

[10] Figliuzzi M, Barrat-Charlaix P, Weigt M (2018). How pairwise coevolutionary models capture the collective residue variability in proteins?. Mol Biol Evol..

[11] Barrat-Charlaix P, Muntoni AP, Shimagaki K, Weigt M, Zamponi F (2021). Sparse generative modeling via parameter reduction of Boltzmann machines: application to protein-sequence families. Phys Rev E.

[12] Xu J (2019). Distance-based protein folding powered by deep learning. Proc Natl Acad Sci.

[13] Greener JG, Kandathil SM, Jones DT (2019). Deep learning extends de novo protein modelling coverage of genomes using iteratively predicted structural constraints. Nat Commun.

[14] Senior AW, Evans R, Jumper J, Kirkpatrick J, Sifre L, Green T, Qin C, Žídek A, Nelson AW, Bridgland A (2020). Improved protein structure prediction using potentials from deep learning. Nature.

[15] Yang J, Anishchenko I, Park H, Peng Z, Ovchinnikov S, Baker D (2020). Improved protein structure prediction using predicted interresidue orientations. Proc Natl Acad Sci.

[16] Russ WP, Figliuzzi M, Stocker C, Barrat-Charlaix P, Socolich M, Kast P, Hilvert D, Monasson R, Cocco S, Weigt M, Ranganathan R (2020). An evolution-based model for designing chorismate mutase enzymes. Science.

[17] Figliuzzi M, Jacquier H, Schug A, Tenaillon O, Weigt M (2016). Coevolutionary landscape inference and the context-dependence of mutations in beta-lactamase tem-1. Mol Biol Evol.

[18] Hopf TA, Ingraham JB, Poelwijk FJ, Schärfe CP, Springer M, Sander C, Marks DS (2017). Mutation effects predicted from sequence co-variation. Nat Biotechnol.

[19] Ackley DH, Hinton GE, Sejnowski TJ (1985). A learning algorithm for Boltzmann machines. Cogn Sci.

[20] Hinton GE (2002). Training products of experts by minimizing contrastive divergence. Neural Comput.

[21] Jaynes ET (1957). Information theory and statistical mechanics. Phys Rev.

[22] Gao C-Y, Zhou H-J, Aurell E (2018). Correlation-compressed direct-coupling analysis. Phys Rev E..

[23] Morcos F, Pagnani A, Lunt B, Bertolino A, Marks DS, Sander C, Zecchina R, Onuchic JN, Hwa T, Weigt M (2011). Direct-coupling analysis of residue coevolution captures native contacts across many protein families. Proc Nat Acad Sci..

[24] Metropolis N, Rosenbluth AW, Rosenbluth MN, Teller AH, Teller E (1953). Equation of state calculations by fast computing machines. J Chem Phys.

[25] Hastings WK (1970). Monte carlo sampling methods using markov chains and their applications. Biometrika.

[26] Geman S, Geman D (1984). Stochastic relaxation, Gibbs distributions, and the Bayesian restoration of images. IEEE Trans Pattern Anal Mach Intell.

[27] Duchi J, Hazan E, Singer Y. Adaptive subgradient methods for online learning and stochastic optimization. In: COLT 2010 - the 23rd conference on learning theory, 2010;pp 257–269

[28] Darken C, Moody J. Note on learning rate schedules for stochastic optimization. In: Proceedings of the 3rd International Conference on Neural Information Processing Systems. NIPS’90, pp. 832–838. Morgan Kaufmann Publishers Inc. 1990.

[29] Ferguson AL, Mann JK, Omarjee S, Ndung’u T, Walker BD, Chakraborty AK. Translating HIV sequences into quantitative fitness landscapes predicts viral vulnerabilities for rational immunogen design. Immunity. 2013;38(3):606–17. 10.1016/j.immuni.2012.11.022. 10.1016/j.immuni.2012.11.022 PMC3728823 23521886

[30] Haldane A, Flynn WF, He P, Vijayan RSK, Levy RM. Structural propensities of kinase family proteins from a potts model of residue co-variation. Protein Science. 2016;1378–1384. 10.1002/pro.2954. 10.1002/pro.2954 PMC4972195 27241634

[31] Haldane A, Levy RM (2021). Mi3-GPU: MCMC-based inverse ising inference on GPUs for protein covariation analysis. Comput Phys Commun.

[32] Bitzek E, Koskinen P, Gähler F, Moseler M, Gumbsch P (2006). Structural relaxation made simple. Phys Rev Lett.

[33] Ekeberg M, Lövkvist C, Lan Y, Weigt M, Aurell E (2013). Improved contact prediction in proteins: using pseudolikelihoods to infer Potts models. Phys Rev E.

[34] Cuturello F, Tiana G, Bussi G. Assessing the accuracy of direct-coupling analysis for RNA contact prediction. RNA. 2020;074179–119. 10.1261/rna.074179.119. 10.1261/rna.074179.119 PMC7161351 32115426

[35] Sarti E, Pagnani A. Infernet-h2020/pfam\_interactions: Initial Release. 10.5281/zenodo.4080947

[36] https://www.ncbi.nlm.nih.gov/

[37] Decelle A, Furtlehner C, Seoane B. Equilibrium and non-equilibrium regimes in the learning of restricted Boltzmann machines. 2021. arXiv:2105.13889

